# Alcohol Consumption Patterns and Sociodemographic Profile in The Tunisian general population

**DOI:** 10.1192/j.eurpsy.2026.10854

**Published:** 2026-07-31

**Authors:** I. Mannoubi, M. Turki, I. Fsili, N. Messedi, M. A. Megdiche, N. Halouani, S. Ellouze, J. Aloulou

**Affiliations:** Psychiatry B, Hedi Chaker hospital, Sfax, Tunisia

## Abstract

**Introduction:**

Alcohol consumption is highly prevalent worldwide, and constitutes a critical public health concern, with patterns largely determined by sociodemographic variables.

**Objectives:**

This study aimed to examine the relationship between alcohol consumption and sociodemographic characteristics in the Tunisian population.

**Methods:**

We conducted a descriptive and analytical cross-sectional observational study was among 161 participants using an anonymous online questionnaire (Google Forms). Individuals aged 18 years or older who provided informed consent were included, while those with incomplete or inconsistent responses were excluded. Collected data included age, sex, marital status, educational level, professional status, socioeconomic level, and place of residence. The “Alcohol Use Disorders Identification Test” (AUDIT) was used to screen for alcohol consumption.

**Results:**

The sample was predominantly female (70.8%), with a mean age of 30 years, mostly living in urban areas (98.8%) and having a university-level education (96.9%). More than half had regular employment (57.8%), and 67.1% were single. Among alcohol consumers (N=63), the mean AUDIT score was 6.27 ± 5.7, with 15.9% meeting criteria for alcohol dependence. Alcohol dependence was significantly associated with younger age (23.7 vs 30.2 years; p=0.041). AUDIT scores were significantly higher among males (4.38 vs 1.67; p=0.006), divorced participants (p=0.0001), those with a primary educational level (p<0.001), unemployed ones (p=0.003), and those with a low socioeconomic status (p<0.001).

**Conclusions:**

This study showed that alcohol consumption in the Tunisian population seems to be strongly influenced by sociodemographic factors. Younger age, male sex, lower educational level, unemployment, divorce, and low socioeconomic status were associated with greater risk of dependence. These findings emphasize the need for targeted prevention and intervention strategies addressing vulnerable groups to reduce the burden of alcohol-related harm.

**Disclosure of Interest:**

None Declared

